# Duplex kidney formation: developmental mechanisms and genetic predisposition

**DOI:** 10.12688/f1000research.19826.1

**Published:** 2020-01-06

**Authors:** Vladimir M. Kozlov, Andreas Schedl

**Affiliations:** 1 iBV, Institut de Biologie Valrose, Equipe Labellisée Ligue Contre le Cancer, Université Cote d’Azur, Centre de Biochimie, UFR Sciences, Parc Valrose, Nice Cedex 2, 06108, France

**Keywords:** CAKUT, kidney development, duplex systems, ureter budding

## Abstract

Congenital abnormalities of the kidney and urinary tract (CAKUT) are a highly diverse group of diseases that together belong to the most common abnormalities detected in the new-born child. Consistent with this diversity, CAKUT are caused by mutations in a large number of genes and present a wide spectrum of phenotypes. In this review, we will focus on duplex kidneys, a relatively frequent form of CAKUT that is often asymptomatic but predisposes to vesicoureteral reflux and hydronephrosis. We will summarise the molecular programs responsible for ureter induction, review the genes that have been identified as risk factors in duplex kidney formation and discuss molecular and cellular mechanisms that may lead to this malformation.

## Introduction

The urinary tract, composed of the kidneys, ureters, bladder and urethra, represents the main excretory system of the mammalian organism. Development of the urinary system, made up of more than 40 different cell types, needs to proceed in a highly organised manner. Given this complexity, it is not surprising that mutations in developmental genes can lead to a wide variety of abnormalities that are usually grouped together as congenital abnormalities of the kidneys and urinary tract (CAKUT). Defects affecting the kidneys range from renal agenesis (a complete lack of kidney development) to hypoplasia (reduced size), dysplasia (abnormally developed tissue), cystic dysplasia, and terminal differentiation defects. Lower urinary tract malformations include vesicoureteral reflux (VUR), hypospadias (opening of the urethra at the lower side of the penis) and posterior urethral valves that often lead to outflow obstructions. Although individual malformations are considered rare diseases, CAKUT, taken together, have an incidence of about 3 to 6 in 1000 live births and thus belong to the most frequent abnormalities detected in the new-born child ^1^. An in-depth presentation of all subclasses and their aetiology would be far beyond the scope of this review and therefore interested readers are referred to other publications that present an overview of CAKUT phenotypes and the genetics underlying them ^2– 6^. Here, we will instead concentrate on duplex (or multiplex) kidneys, a very frequent subclass of CAKUT, which is often neglected in the literature.

## Development of the urinary system

To understand the aetiology of duplex kidneys, it is important to consider how the urinary system forms. From a developmental point of view, the urogenital tract derives from two independent germ layers with kidneys and ureters arising from the intermediate mesoderm (IM) and the bladder and urethra developing from cloacal endoderm ^7^. Accordingly, malformations of the urinary system can be further classified into congenital abnormalities of the upper and lower urinary tract (and the latter are sometimes abbreviated as CALUT). Despite this developmental distinction, it should be noted that some authors group malformations of the ureter as part of congenital abnormalities of the lower urinary tract.

Kidney development in mammals commences with the formation of the nephric duct (ND) at the anterior (rostral) pole of the IM. As development proceeds, epithelial cells of the ND proliferate and actively migrate towards the caudal end of the nephrogenic cord ^8– 10^. Eventually, the ND fuses with the cloaca, a process that involves dedicated apoptosis and requires GATA3 and LHX1 as well as retinoic acid and RET and FGF signalling ^9– 13^.

As the ND elongates caudally, a series of tubules forms within the nephrogenic cord. The most anteriorly positioned pronephric tubules are considered an evolutionary remnant and are non-functional in mammals. Subsequently, a wave of mesonephric tubules develop that fall into two groups. While rostrally positioned tubules are connected to the ND and serve as an embryonic kidney, more caudally located tubules do not drain into the ND and are non-functional ^14, 15^. Both pronephros and mesonephros are transitory structures in the mammalian embryo and disappear (pronephros) or are remodelled (mesonephros) at later stages of development.

The metanephros represents the permanent kidney in mammals and develops at the most caudal position of the IM. Metanephros development is first detectable as a population of slightly condensed mesenchymal cells within the nephrogenic cord which express a set of molecular markers ( *HOX11*, *SIX2*, *GDNF*, *EYA1*) ^15, 16^. In normal development, signalling from the metanephric mesenchyme (MM) induces the formation and outgrowth of a single ureteric bud (UB) from the ND, which will invade the MM and undergo a first stereotypic dichotomous branching event (T-shaped ureter). The collecting duct system (ureteric tree) forms through further rounds of branching that often include tri-tips, which, however, eventually resolve into ureter bifurcations ^17, 18^. In return, signals released from the ureter will induce the MM to differentiate into nephrons, the functional units of the kidney. For further details on this process, we refer the reader to recent reviews ^19– 22^.

Development of the urinary system is not restricted to kidney formation but also involves extensive developmental remodelling of the lower tract. An excellent and detailed description of this complex process can be found in 7. In brief, the emerging UB is initially connected to the cloaca via the distal part of the ND, also termed the common nephric duct (CND). Downgrowth of the urorectal septum leads to a separation of the cloaca into a ventrally located urogenital sinus and a dorsally positioned anorectal sinus ^7, 23– 25^. The cranial urogenital sinus will further elongate to develop into the bladder, whereas its posterior portion will form the urethra. As development proceeds, apoptosis eliminates the CND, leading to the fusion of the ureter with the future bladder, thus creating the ureterovesical junction ^26^.

## Classification and epidemiology of duplex kidneys

**Figure 1.**
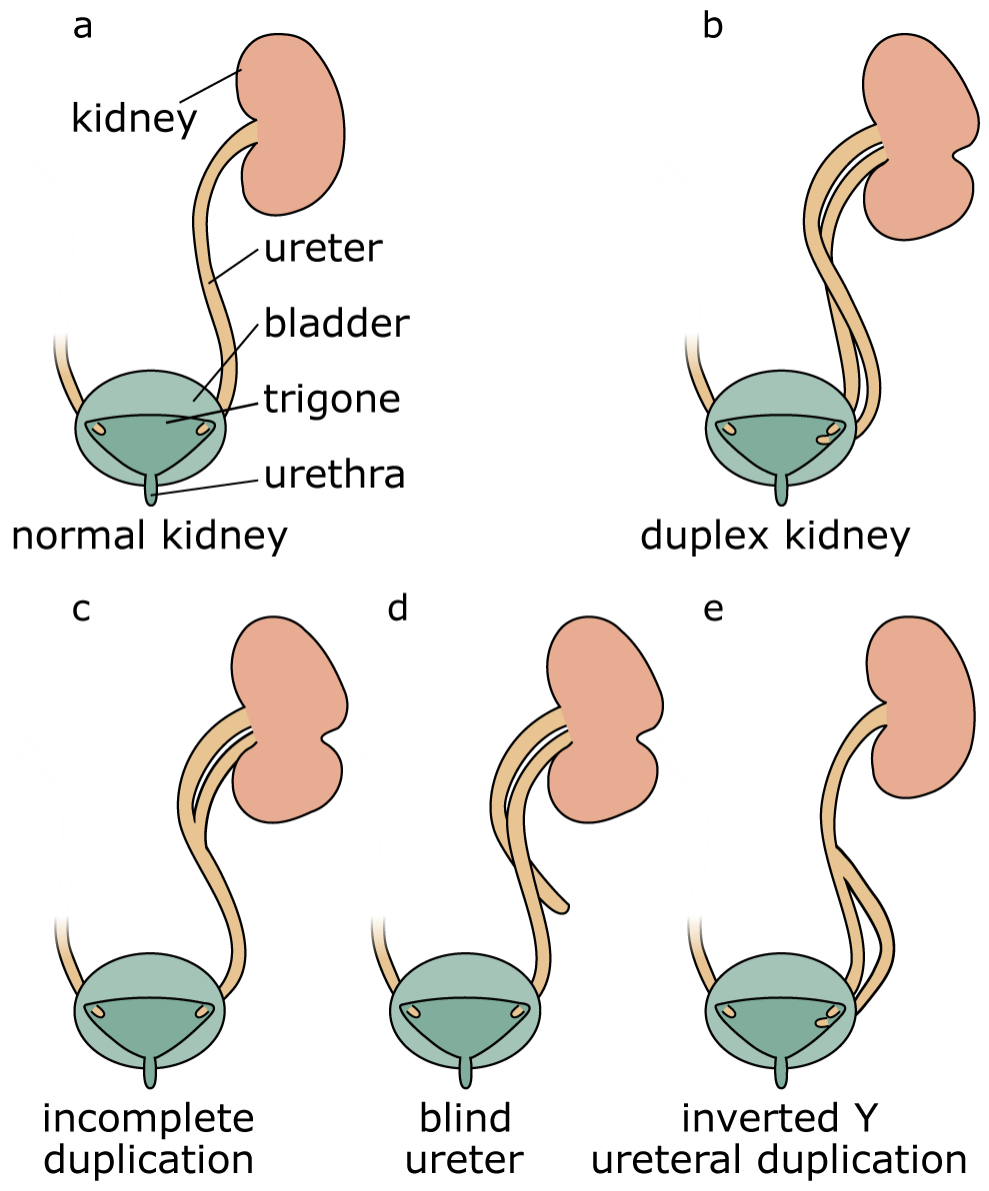
Classification of duplex kidney anatomy. Compared with a normal kidney ( **a**), complete duplication produces a duplex kidney with two poles that drain into two ureters ( **b**). Incomplete duplication leads to a Y-shaped ureter ( **c**). Blind ureters do not drain into the bladder ( **d**). In the rare case of inverted Y-ureteral duplication, two ureters fuse before entering the kidney ( **e**).

Duplex systems can have a variety of phenotypes, and multiple classification systems have been proposed to categorise this pathology ( Figure 1) ^27^. In incomplete duplication, the two poles of a duplex kidney share the same ureteral orifice of the bladder. Such duplex kidneys with a bifid pelvis or ureter arise when an initially single UB bifurcates before it reaches the ampulla. This is likely caused by a premature first branching event that occurred before the ureter has reached the MM. Much more frequent are complete duplications, which occur when two UBs emerge from the ND. In most cases, the lower pole of the kidney is normal and the upper pole is abnormal ^28, 29^, an observation explained by the fact that the ectopic UB frequently emerges anteriorly to the position of the normal UB and drives the formation of the upper pole of a duplex kidney. Inverted Y-ureteral duplication is a rare condition in which two ureteral orifices drain from a single normal kidney. Inverted Y-ureteral duplication is believed to be caused by the merging of two independent UBs just before or as they reach the kidney anlagen ^30^. A very rare H-shaped ureter has also been reported ^31^. Although the vast majority of cases involve a simple duplication, multiplex ureters with up to six independent buds have also been described ^32– 37^. In some cases, the additional ureter or ureters are ectopic and fail to connect to the bladder or the kidney (blind ending ureter) ^33^.

**Figure 2.**
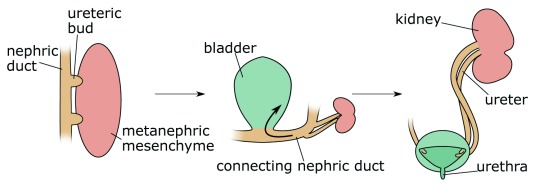
Morphogenesis of duplex kidney. Duplex kidneys form through the induction of two ureteric buds from the nephric duct that will invade the metanephric mesenchyme. Subsequently, apoptosis of the common nephric duct (CND) leads to the insertion of both ureters into the developing bladder with the orifice of the initially posteriorly positioned ureteric bud ending up in a superior position.

The aetiology of most duplex kidneys can be traced back to the very first induction steps of the ureter. In the majority of cases, an additional UB emerges in a rostral position to the normal outgrowth. By contrast, in adults, the upper (abnormal) kidney pole drains into the bladder at a site distal to the orifice of the lower kidney pole ^38^. This paradoxical phenomenon, known as the Weigert–Meyer rule ^29^, can be explained by the significant amount of remodelling occurring at the future ureter–bladder junction during development. Indeed, as apoptosis eliminates the CND, the ureter inserts into the developing bladder and moves upwards ( Figure 2) ^7, 39– 41^. An initially anteriorly positioned ureter thus ends up with a more distal insertion site in the bladder, a model that has been proposed by Mackie and Stephens ^42^. Correct positioning of the ureter into the bladder is important to allow formation of a normal trigone (the triangle formed by the two ureter orifices and the urethra) and prevent ureter reflux caused by a malfunctioning valve or a too-short ureter tunnel. Because the vast majority of duplex kidneys arise from an ectopic bud in a rostral position, it is usually the upper pole of the kidney that is affected by VUR and hydronephrosis.

Estimates suggest a prevalence of duplex kidneys of between 0.2 and 2% in the general population, and females are affected twice as frequently as males ^38, 43^. The reasons for this sex bias are unknown. About 40% of patients with duplex kidneys have been reported to exhibit pathological manifestations ^43^. However, because duplex kidneys are frequently asymptomatic and therefore predominantly detected in patients who seek medical assistance, the actual percentage of patients with symptoms is likely to be lower. Symptoms associated with duplex kidneys can include pain, haematuria, dysuria and difficulty or abnormal frequency of micturition ^38, 43^. Specific manifestation of the pathology depends on the anatomy of each duplication event ^44^. Furthermore, duplex kidneys are linked to a number of renal disorders, including pelvi-calyceal dilatation, cortical scarring, VUR, hydronephrosis, ureterocoeles on the non-duplex side, caliculi or yo-yo reflux (in the incomplete duplication cases) ^38, 43^.

## Molecular pathways controlling ureter induction

**Figure 3.**
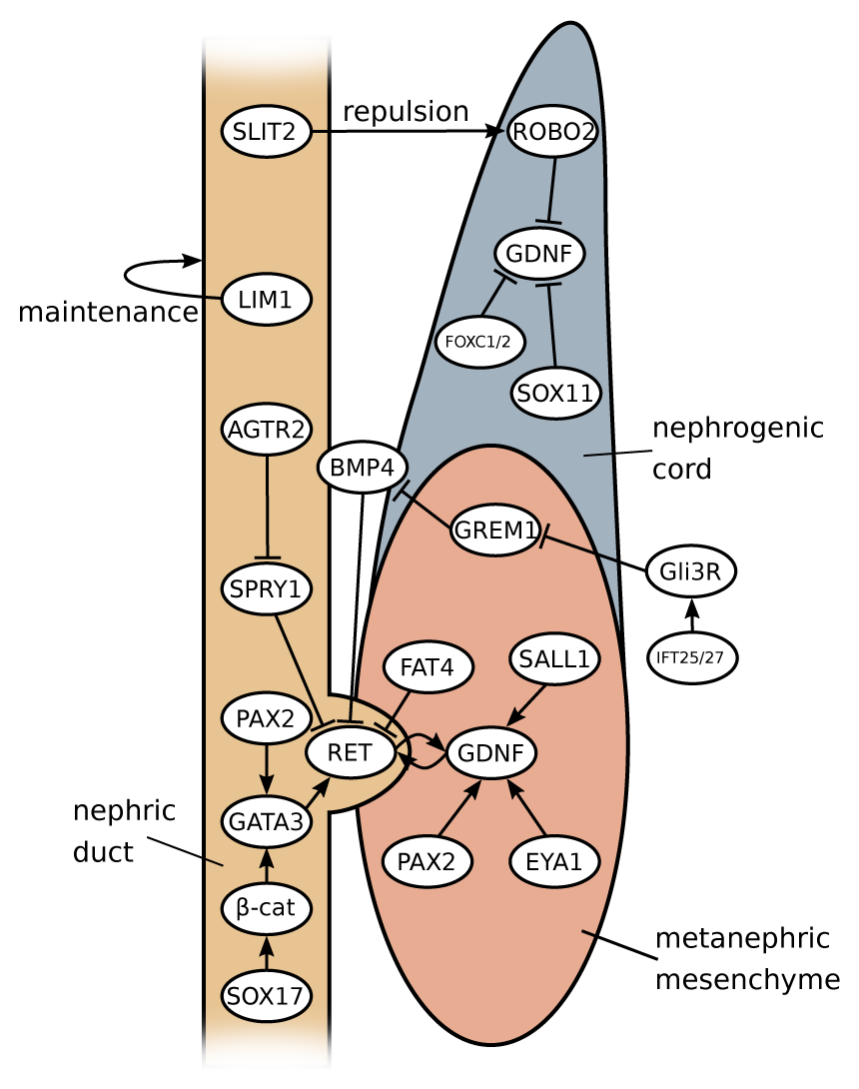
Molecular interactions during kidney development. GDNF-RET signalling is at the core of the signalling network in kidney development and is responsible for ureteric bud (UB) emergence. GDNF expression is positively modulated by factors expressed in metanephric mesenchyme (PAX2, EYA1 and SALL1) and negatively (probably in an indirect manner) by SOX11, FOXC1, FOXC2 and ROBO2 in anterior domains of the nephrogenic cord. Ectopic formation of the UB is prevented by the factors expressed in nephric duct (SLIT2, SPRY1 and GATA3) and in the enveloping mesenchyme (BMP4 and FAT4). Influence from other upstream factors leads to the formation of a complex regulatory landscape.

If duplex kidney formation is rooted in the formation of two ureteric tips, how can we explain the outgrowth of supernumerary buds on a molecular level? Interactions between MM and the ND are crucial to ensure the induction of the ureter from the ND, and a key pathway controlling this process is the GDNF/RET signalling axis ( Figure 3) ^45^. GDNF, a distant member of the transforming growth factor beta (TGFβ) superfamily of signalling molecules, is specifically expressed within the MM, whereas its cognate receptor RET is expressed along the entire length of the ND. Binding of GDNF to RET is greatly facilitated by the co-receptor GFRα1. The requirement for these genes in ureter outgrowth has been extensively demonstrated by using gene targeting in mice, and homozygous mutations in either of these genes leads to a failure of ureter induction and consequently renal agenesis ^46– 50^. Binding of GDNF to the receptor tyrosine kinase RET induces autophosphorylation and recruitment of the tyrosine phosphatase SHP2 ^51, 52^, which results in the activation of several intracellular signalling cascades, including RAS/MAPK, PLCγ/Ca ^2+^, PI3K-AKT ^53^, and culminates in the transcriptional activation of a set of downstream target genes ^54^. Ureter branching appears to involve, in particular, the ERK/MAPK pathway, and mice lacking the kinases *Mek1* and *Mek2* fail to form a properly branched ureteric tree ^55^. Activated RET signalling induces not only proliferation but also cellular motility. Indeed, experiments in chimeric mice demonstrated that wild-type cells move towards the tip of the UB but that *Ret* mutant cells are left behind ^56^. This cellular sorting mechanism ensures a strong and directed response that, under normal circumstances, results in the outgrowth of a single UB.

### Factors regulating Gdnf expression

Given the crucial function of *Gdnf* in ureter induction, we need to consider how the expression of this gene is regulated. Activation of *Gdnf* in the mesenchyme relies on a set of transcription factors, including SALL1, PAX2 ** and ** EYA1. ** Deletion of either of these factors in mice leads to a lack of ureter induction and consequently to renal agenesis ^57– 59^ *.* Heterozygous mutations in each of these genes have been shown to be involved in CAKUT ^5, 6^, and SALL1, in particular, has also been linked to duplex kidney formation ^60^. In addition, *Gdnf* mRNA levels appear to be regulated post-transcriptionally via its 3’ untranslated region (UTR). Indeed, replacement with a heterologous UTR sequence resulted in increased *Gdnf* expression levels that were associated with ND remodelling defects independent of apoptosis ^61^.

In the mouse, *Gdnf* expression commences in rostral domains of the nephrogenic cord at embryonic day 9.5 (E9.5), about 1 day before ureter induction. The ND, however, does not respond to the GDNF signal in those anterior regions and this is likely to be due to two reasons: First, the anterior IM has relatively high levels of BMP signalling, which is known to suppress ureter branching (see the ‘Restricting Ret activation’ section below). Second, SLIT/ROBO signalling, a pathway that is known for its role in axon repulsion ^62^, appears to repulse *Gdnf*-expressing cells from the ND, thus causing a physical separation of these two structures in anterior regions ^63^. *Robo2* knockout mice lack this separation and show ectopic buds along the entire length of the ND ^64^. The physical separation of ND and *Gdnf*-expressing cells may explain why in *Foxc1*, *Foxc2* and *Sox11* mouse mutants, which all display a dramatic expansion of *Gdnf* expression, the ND does not respond in the anterior domain ^64– 66^. Instead, only the region just rostrally to the normal site of induction responds by forming a second ureter. Mutations in *ROBO2*, *SLIT2* and its associated GTPase-activating protein *SRGAP2* are found in patients with VUR and duplex systems ^67, 68^.

**Figure 4.**
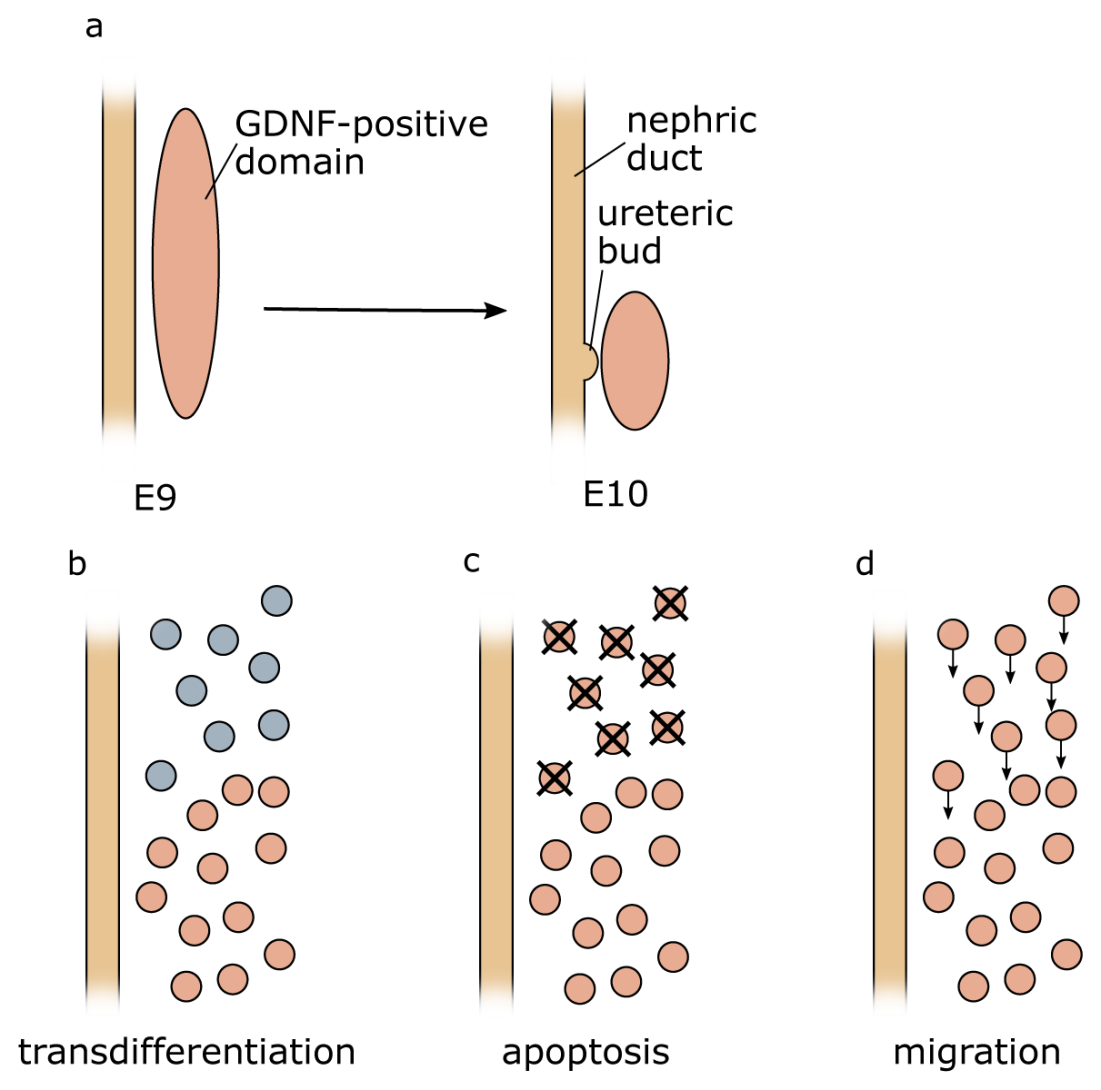
Possible causes of caudal *Gdnf* domain restriction. ( **a**) At early stages during development, *Gdnf* expression can be found in rostral domains of the intermediate mesoderm but over time becomes caudally restricted. Three mechanisms could explain this observation: ( **b**) active suppression of *Gdnf* expression in more rostral domains ( **c**), apoptosis of *Gdnf-*expressing cells ( **d**), or migration of the cells towards the caudal end of the intermediate mesoderm. E, embryonic day.

By the time of ureter induction (E10.5 in mice), mesenchymal cells that express *Gdnf* become restricted to the caudal part of the MM ( Figure 4). Three possible mechanisms for this restriction could be envisaged: (1) Active suppression of *Gdnf* expression in more rostral domains could occur. Since the expression of *Foxc1* and *Sox11* overlaps with the *Gdnf* domain, active suppression of the latter seems unlikely. (2) *Gdnf*-positive cells at the rostral end could undergo cell death. The pro- and mesonephros are known to be subject to massive apoptosis, although this seems to affect, in particular, the epithelial cells of tubules. However, preliminary data from our lab suggest that mesenchymal cells positioned just rostrally of the *Gdnf*-expressing domain also undergo apoptosis. (3) Finally, rostrally positioned *Gdnf*-positive cells may undergo directed migration towards the caudal end. The proposed distinct origin of ND and MM from the anterior and posterior IM, respectively, would argue against this possibility ^16^. However, *Slit/Robo* signalling and members of the *SoxC* class genes have been implicated in cell migration ^62, 69^. Perhaps *Gdnf* restriction is a combination of several mechanisms, including cell clearance through apoptosis and directed cell migration of anteriorly positioned *Gdnf*-positive cells. A careful analysis of mouse mutants showing an expansion of the *Gdnf* expression domain, perhaps coupled with live imaging in explant cultures, may help to address this open question.

### Pathways in nephric duct–specific activation

PAX2 not only is involved in the activation ** of *GDNF* but also is required for the expression of ND-specific genes. A key target appears to be the transcription factor GATA3, which in turn transcriptionally activates *Ret*. Tissue-specific knockout mice that lack *Gata3* within the ND show an altered response to local growth factors (GDNF and FGF) and display premature cell differentiation and differential cell adhesion properties. As a result, cells with sufficient levels of GATA3 and RET segregate from GATA3-deficient cells and expand, forming ectopic buds and kidneys ^70^.

Beta-catenin, a multifunctional protein involved in cell–cell adhesion and transcriptional regulation, appears to be one of the factors involved in this growth. Conditional inactivation of β-catenin in the ND leads to a range of kidney defects, including duplex kidney formation ^71^. Molecular markers affected in these mutants were the transcription factors EMX2 and SOX9, both of which are known to be involved in ureter budding ^72, 73^. However, ectopic budding was observed only in cases where loss of β-catenin expression was mosaic ^74^. Hypoxia-induced reduction of β-catenin has also been shown to cause duplex kidneys amongst other CAKUT phenotypes ^75^. Beta-catenin action during kidney induction is mediated at least partly through the transcription factor GATA3 ^70^.

*Sox17* mutations have been identified in a cohort of human patients with CAKUT, including a duplicated pyeloureteral system. The authors demonstrated that the mutation influenced protein stability and reduced β-catenin activity ^76^. It is therefore possible that the mutated SOX17 protein leads to lower β-catenin and, in turn, reduced GATA3 levels.

In parallel to GATA3, LHX1 (LIM1) appears to be essential in permitting normal budding ^77^. Tissue-specific deletion of LIM1 in ND derivatives leads to renal hypoplasia and hydronephrosis and an impaired extension of the ND. Some conditional mutants of *Lim1* also display incomplete duplication of kidney ureters where both poles of a duplex kidney merge before entering the bladder. This form of duplex kidney was traced back to the first UB branching event, where defective UB forms a Y-shaped rather than a T-shaped structure ^9^.

### Restricting Ret activation

To limit ureter outgrowth to a single site, a series of negative regulators that suppress the RET signalling cascade are in place. BMP signalling, in particular, seems to be a suppressor of ureter outgrowth and branching, and heterozygous *Bmp4* mutations in mice lead to a wide range of CAKUT phenotypes, including duplex kidneys ^78^. BMP and FGF signalling are known antagonists in epithelial branching of the lung ^79^ but also kidney development ^80^. Since FGF and RET receptors are receptor tyrosine kinases that use similar intracellular signal transduction pathways, we can reason that the antagonistic action of BMP acts in analogous fashion on RET signalling. To permit ureter outgrowth specifically at the site of the future kidney, MM cells express the BMP inhibitor Gremlin ( *Grem1*), which counteracts the BMP function ^81^. Heterozygous *BMP4* and *GREM1* mutations have both been identified in human patients with CAKUT ^82, 83^, although it is not clear whether variants in these genes also predispose to duplex kidney formation.

A number of other genes involved in duplex kidney formation appear to affect the BMP/Gremlin axis. Mutants for the intraflagellar transport proteins IFT25 or IFT27, which are believed to increase GLI3R, a repressor of SHH signalling, show a high penetrance of duplex kidney formation (~50%) ^84^. Similarly, constitutive expression of a truncation mutation in *Gli3* ( *Gli3* ^Δ699^), which is found in Pallister–Hall syndrome and is likely to sensitise tissue for SHH signalling, causes CAKUT with duplex kidneys ^85^. The phenotype has been linked to an increased sensitivity of the ND by lowering BMP4 signalling.

Of interest, several genes that are implicated in the formation of cilia (for example, *Cep290*, *Dync2h1*, *Tbc1d32* and *Tmem67*) have also been implicated in duplex kidney formation ^86^. The primary cilia is an organelle that has a key function in cellular signalling ^87^, and SHH signalling, in particular, is directly linked to this organelle. Because SHH signalling has been proposed to be involved in duplex kidney formation ( 88 and above), it is tempting to speculate that the above cilia-related genes also influence this pathway.

In addition to extracellular modulators, cytoplasmic antagonists exist to suppress ureter outgrowth. Most notably, Sprouty ( *Spry1*) suppresses MAPK signalling in the absence of GDNF, and inactivation in mice results in the formation of multiple UBs ^89^. Signalling through the angiotensin receptor appears to be important in suppressing *Spry1* expression ^90^ but also in activating *Ret* expression, and *Agtr2* knockout mice show a range of CAKUT phenotypes, including a duplex system ^91^. To date, no pathogenetic *SPRY1* mutations have been identified in patients with CAKUT, and it is currently unclear to what extent this gene contributes to duplex kidney formation in human patients. Interestingly, in the absence of *Spry1*, GDNF signalling is no longer required for ureter induction, and *Spry1 ^-/-^/Gdnf ^-/-^* double knockout mice develop normal kidneys. In this context, FGF10, which normally plays only a minor role in kidney development, becomes indispensable for kidney induction, and ** triple mutants ( *Fgf10 ^-/-^/Spry1 ^-/-^/Gdnf ^-/-^*) display renal agenesis ^92^. FGF signalling thus can be considered a reinforcing signal that contributes to enhanced epithelial growth and budding. FGF signalling serves as the main pathway in branching morphogenesis of other organs such as the lung ^93^, and we can speculate that GDNF/RET signalling has taken over the ancestral function of FGF in epithelial branching of the kidney.

Finally, tissue-specific knockout of *Fat4* within the nephrogenic cord results in a duplex kidney phenotype that can be rescued by reducing the dose of GDNF ( *Gdnf* ^+/-^). Recent molecular experiments demonstrated that FAT4 directly binds to RET and restricts its activity in the ND/UB by disrupting the formation of RET-GFRA1-GDNF complex ^94, 95^.

There are a number of other genes which have been shown to be implicated in duplex kidney formation but for which the molecular events leading to supernumerary buds are not well defined. Because in these cases the causative nature of mutations for duplex kidney formation is less established, we refrain from a mere listing of genes at this place. The interested reader is referred to Table 1 of genes involved, the associated phenotypes and corresponding references.

**Table 1.** Genes involved in duplex kidney formation.

Group	Genotype	Mechanism	Reference
GDNF domain			
	*Robo2 ^-/-^*	Abnormal *Gdnf* expression domain MM fails to separate from WD	Grieshammer *et al.* ^64^ Wainwright *et al*. ^63^
	*Slit2 ^-/-^*	Abnormal *Gdnf* expression domain	Grieshammer *et al.* ^64^
	Foxc1 ^-/-^	MM fails to reduce in size	Kume *et al*. ^65^ Komaki *et al*. ^96^
	*Sox11 ^-/-^*	MM fails to reduce in size	Neirijnck *et al*. ^66^
Increased sensitivity of WD			
	*Bmp4 ^+/-^*	Lack of inhibition of WNT11, a target of GDNF	Miyazaki *et al*. ^78^ Michos *et al*. ^81^
	*Ift25 ^-/-^, Ift27 ^-/-^*	Increased sensitivity of WD through Gremlin-BMP4 cascade	Desai *et al*. ^84^
	*Gli3 ^Δ699/Δ699^*	Increased sensitivity of WD through Gremlin-BMP4 cascade	Blake *et al*. ^85^
	*Agtr2 ^-/Y^*	Disrupted renin-angiotensin signalling leads to aberrant UB morphogenesis	Nishimura *et al*. ^91^ Yosypiv *et al*. ^90^
	*p53 ^-/-^, p53 ^UB-/-^*	Increased response of WD to GDNF signal. Two ureters fuse in the later development and resemble a bifurcation	Saifudeen *et al*. ^97^ El-dahr *et al*. ^98^
	*Fat4 ^-/-^ Fjx1 ^-/-^*	Premature branching with incomplete duplication due to overactive GDNF-RET signalling	Saburi *et al*. ^94^ Zhang *et al*. ^95^
	*Hoxb7-Cre β-catenin ^-/c^*	Ectopic activation of UB branching pathway in WD	Marose *et al*. ^74^
	*Spry1 ^-/-^*	Increased sensitivity of WD to GDNF-RET signalling	Basson *et al*. ^89^
	*Gata3ND ^-/-^*	The entire length on WD is covered by ectopic UBs, most of which subsequently regress	Grote *et al*. ^70^
Cell polarity defect			
	*T-Cre Wnt5af ^l/Δ^*	Double UB, abnormal morphology of posterior WD, defects in IM morphogenesis	Yun *et al*. ^99^
	Ror2 ^-/-^	Similar to *Wnt5a* phenotype	Yun *et al*. ^99^
Cell adhesion defect			
	*L1 ^-/Y^*	Either incomplete or complete duplication. Double UB on WD or accessory budding from the main ureter	Debiec *et al*. ^100^
Unknown			
	*Pax2 ^+/-^*	Premature branching with incomplete duplication, linked with inactivation of GDNF expression	Brophy *et al*. ^101^
	*Pax2-Cre Lim1 ^Δ/Δ^*	WD fails to extend caudally; UB is absent or Y-shaped	Pedersen *et al*. ^9^
	*Cc2d2a, Mks1,* *Cep290, Dync2h1,* *Tbc1d32, Tmem67*	Duplex kidney as a part of a ciliopathy phenotype	San agustin *et al*. ^86^
	*Sox17 ^Y259N/+^*	Duplicated pyeloureteral collecting system	Gimelli *et al*. ^76^
	*Nfia ^-/-^*	Partial ureteral duplication	Lu *et al*. ^102^
	*Adamts18* ^-/-^	Complete ureteral duplication, increased nephron endowment	Rutledge *et al*. ^103^

## Conclusions and perspectives

As we have seen, the seemingly simple event of ureteric budding is a highly complicated and stringently controlled process that employs both positive and negative feedback loops. As such, the fact that a large number of genes are involved in duplex kidney formation is not surprising, and future analyses are likely to identify many more factors involved in these abnormalities. However, the incomplete penetrance of phenotypes and the multigenic basis of this malformation make the confirmation of mutations as being disease-causing increasingly difficult. Indeed, our own unpublished data suggest that duplex kidney phenotypes can be highly genetic background–dependent, indicating the presence of modifier genes. Moreover, intergenic/regulatory mutations or epigenetic mechanisms that affect gene expression levels rather than protein function are likely to contribute to disease. Finally, we should keep in mind that, despite a large degree of conservation, mice and humans show significant differences on a developmental and molecular level ^104– 106^. Findings in knockout mice are therefore only indicative and should not be directly extrapolated to human patients. Future research should also address the disparity in the frequency of duplex kidneys occurring in men and women. In the long run, the integration of a large amount of whole genome sequencing data coupled with a better understanding of how gene regulation is achieved will be required to corroborate the involvement of genomic changes and predict the phenotypic outcome in duplex kidneys.
